# Isolation and characterization of 8 microsatellite loci for the “killer shrimp’’, an invasive Ponto-Caspian amphipod *Dikerogammarus villosus* (Crustacea: Amphipoda)

**DOI:** 10.1007/s11033-014-3742-0

**Published:** 2014-09-19

**Authors:** Tomasz Rewicz, Rémi A. Wattier, Thierry Rigaud, Karolina Bacela-Spychalska, Michal Grabowski

**Affiliations:** 1 Department of Invertebrate Zoology and Hydrobiology, University of Lodz, 12/16 Banacha, 90-237 Lodz, Poland; 2 Equipe Ecologie Evolutive, UMR CNRS 6282 Biogéosciences, Université de Bourgogne, 6 Boulevard Gabriel, 21000 Dijon, France

**Keywords:** Invasive species, Population genetics, *Dikerogammarus villosus*, Biological invasions, Polymorphic loci

## Abstract

*Dikerogammarus villosus* is a freshwater amphipod of the Ponto-Caspian origin recognized as one of the 100 worst alien species in Europe, having negative impact on biodiversity and functioning of the invaded aquatic ecosystems. The species has a wide ecophysiological tolerance and during the last 20 years it has rapidly spread throughout European inland waters. In consequence, it presents a major conservation management problem. We describe eight polymorphic microsatellite loci developed for *D. villosus* by combining a biotin-enrichment protocol and new generation 454GS-FLX Titanium pyrosequencing technology. When genotyped in 64 individuals from two locations, the loci exhibited a mean diversity of 4.87 alleles per locus (2–13). The mean observed and expected heterozygosities were, respectively, 0.439 (0.091–0.844) and 0.468 (0.089–0.843). Gametic disequilibrium was not detected for any pair of loci. The microsatellite markers will be a valuable tool in assessing the demographic processes associated with invasion of the killer shrimp from a genetic point of view.

## Introduction

The Ponto-Caspian amphipod *Dikerogammarus villosus* (Sowinsky, 1894), also known as the killer shrimp, is recognized as one of the 100 worst alien species in Europe [1]. This invader has colonized most of the European main inland water bodies in less than 20 years [2–4]. The threat it poses to ecosystems and species diversity is significant [5]. The killer shrimp is an efficient, high trophic level predator [6], feeding on other amphipods and on almost all other available benthic invertebrates [7, 8]. In addition, this species is characterised by wide ecophysiological tolerance to a number of environmental factors including water temperature, salinity and oxygen concentrations [9–12] as well as by very high fecundity [13–15]. Both features are highly advantageous in colonizing new areas. Initial expansion of *D. villosus* in continental Europe followed the two so-called invasion corridors for Ponto-Caspian fauna, associated with major rivers (i.e. the Southern Corridor via Danube/Rhine and the Central Corridor via Dnieper/Vistula) often referred to as ”invasion highways” [2]. The populations migrating via the two invasion corridors originating in different Ponto-Caspian watersheds are about to come into contact in Poland [4] and possibly hybridize. Further expansion of the killer shrimp is currently in progress. It has recently colonized many lakes in the Alpine region [16] and was even accidentally introduced overseas to the UK [17]. Finally, the risk of its future introduction to the North American Great Lakes is not negligible.

The microsatellite markers will be a valuable tool in assessing the demographic processes associated with invasion of the killer shrimp from a genetic point of view. For example, they will help to identify the origin of populations in the UK and in Alpine lakes as well as to assess the dynamics of the invasion process (e.g. via the associated bottleneck or founder effect). Such marker will also help to estimate the differentiation between invasion corridors and chances for putative hybridization in case the two populations originating in different areas of the native range (Danube vs. Dnieper) meet in Poland. The three already known loci [18] proved to be useful [19] but additional loci are needed to answer more detailed questions.

## Materials and methods

The total genomic DNA from eight *D. villosus* individuals was extracted with standard phenol–chloroform method. Enrichment for eight microsatellite motifs [i.e. (AG)_10_, (AC)_10_, (AAC)_8_, (AGG)_8_, (ACG)_8_, (AAG)_8_, (ACAT)_6_, (ATCT)_6_] was based on a biotin protocol adapted from Kijas et al. [20]. The sequences were produced by pyrosequencing on a 454 GS-FLX Titanium^®^ apparatus (Roche Diagnostics). Both, the enrichment and the pyrosequencing were as described by Malausa et al. [21]. Using the open access QDD program, the resulting 32,084 sequences were first screened for microsatellite (minimum of five repeats) and flanking sequences presence and then PCR primers were designed for selected sequences [22]. From a total of 4,206 candidate sequences including microsatellites, the primer design was effective for 102 putative loci. All the steps from enrichment down to primer design were performed at Genoscreen ^®^ (Lille, France). Thirty-three primer pairs were selected for amplification. Each forward primer was 5′ tailed with a M13 sequence (5′-AGGGTTTTCCCAGTCACGACGTT-3′). The PCRs were carried out in a 10 µl volume including 20 ng DNA template, 200 nM each primer (Table 1), 0.025 µM of 5′ labeled M13 primer (either 700 or 800 dye), 5 µl DreamTaq Master Mix (2x) DNA Polymerase (Thermo Scientific). The reactions were run in a BioRad thermocycler with an initial denaturation step at 95 °C for 3 min, followed by 35 cycles consisting of 20 s at 95 °C, 45 s at 50 °C and 1 min at 72 °C, and a final extension step at 72 °C for 2 min. Product size variations was visualized with the LICOR 4200L automated sequencer. The polymorphism was tested on seven individuals from five locations in Europe: Liman Duru Golu, Turkey (41.316N; 28.621E); Danube delta, Ukraine (45.337N; 28.955E); Dnieper mouth, Ukraine (47.792N; 35.126E); Grafham water, UK (52.292N; −0.324W); Constance Lake, Germany (47.748N; 9.137E). From the 33 microsatellite loci chosen for amplification, ten failed to produce readable patterns, fifteen loci were monomorphic and eight primer pairs revealed polymorphism Further, the allelic diversity of the eight candidate loci was tested on 64 individuals, from the Danube delta in Ukraine (DAN; n = 32) and from the Dnieper mouth in Ukraine (DNI; n = 32). These two populations may be considered as representatives of the two distinct watersheds areas in the Ponto-Caspian region providing starting points for the killer shrimp invasion. The allelic diversity, observed (Ho) and expected (He) heterozygosities, deviations from Hardy–Weinberg proportions as well as gametic disequilibrium and differentiation between DAN and DNI (Fst as estimated by Weir and Cockerham Theta) were estimated using the software Fstat version 2.9.3.2 [23]. When appropriate, the comparisons included Bonferroni correction for multiple tests. Presence and possible source of genotyping errors (null allele, stuttering, short allele dominance, [24] were checked with Micro-checker version 2.2.3. [25].

**Table 1:** Characterization of 8 polymorphic microsatellite loci for *Dikerogammarus villosus*

Locus	Repeat motif	Primer sequence (5′-3′)	Genbank Accession#	Size range (bp)	*Pop*	*N*	*K1*	*K2*	*H*_*o/*_ *H*_*e*_	*Fis*	*Null*
Dv1-F842 K	(AC)_7_	F:CAATGGGTGACACATCGAGA	GF112174	170–178	DAN:	25	3	2	0.120/0.246	0.517	0.097
		R: GCTCGGCTGCTTGTTTTATT	–		DNI:	27		2	0.185/0.372	0.508	0.132
Dv6-GQL0 M	(CA)_7_	F: ACACTGCCTATGTTTCCCCA	GF112181	150–190	DAN:	20	6	6	0.400/0.605	0.345	0.119
		R: AGGAAGCAAGGATTTAGGGC	–		DNI:	31		4	0.419/0.654	0.362	0.136
Dv11-cons108	(TG)_7_	F: ATATGTCTGAGAGCATTTTGCC	GF112175	190–194	DAN:	26	3	3	0.538/0.664	0.193	0.068
		R: GTCGGTAAATCGACGCAT	–		DNI:	27		2	0.704/0.507	−0.399	−0.137
Dv13-F64EY	(GT)_8_	F: TCCATCAGGTGTTAACCAGTACA	GF112176	205–215	DAN:	31	4	4	0.613/0.569	−0.079	−0.034
		R: TGGGGTTTCCGTATTTGTCT	–		DNI:	32		3	0.281/0.250	−0.130	−0.028
Dv17-GP5PA	(GT)_10_	F: CCTTTATATGCGAAAAGCCG	GF112177	178–192	DAN:	30	6	4	0.467/0.525	0.114	0.033
		R: CCTGGAGTTGAAATGAGACACA			DNI:	31		4	0.484/0.411	−0.181	−0.056
Dv19-FQPCJ	(CAA)_6_	F: GAATTTCGAATCAATTTCCCC	GF112178	88–90	DAN:	22	2	2	0.091/0.089	−0.024	−0.003
		R: GGAGCATGAGGCCAAGTAAA	–		DNI:	32		2	0.625/0.458	−0.372	−0.119
Dv31-cons60	(TGT)_10_	F: TTTCGAAAGGGGTGAAAATTA	GF112179	121–124	DAN:	32	2	2	0.188/0.268	0.303	0.060
		R: AATAGCACAGACCGCTCGAC	–		DNI:	32		2	0.281/0.289	0.028	0.003
Dv33-cons89	(TAGGT)_15_	F: TTACAGGATGCCGAATACCA	GF112180	155–235	DAN:	32	13	10	0.844/0.843	−0.001	−0.007
		R: TTACAAATCCAATATAACCTTGGC	–		DNI:	30		10	0.781/0.730	−0.071	−0.038

*Pop* sampled populations; *DAN* Danube delta in Ukraine; *DNI* Dnieper mouth in Ukraine; *N* number of successfully genotyped individuals; *K1* and *K2* number of alleles for both populations combined and in each population respectively; *H*_*o*_ and *H*_*e*_ observed and expected heterozygotes; *Fis* standarised genetic variance within populations at each locus; *Null* frequency of null allele as measured by Brookfield1 method in Micro-checker

## Results and discussion

Out of the 33 microsatellite loci chosen for testing, ten did not amplify at all, 15 were monomorphic and eight amplified successfully and revealed polymorphism.

**Fig. 1:**
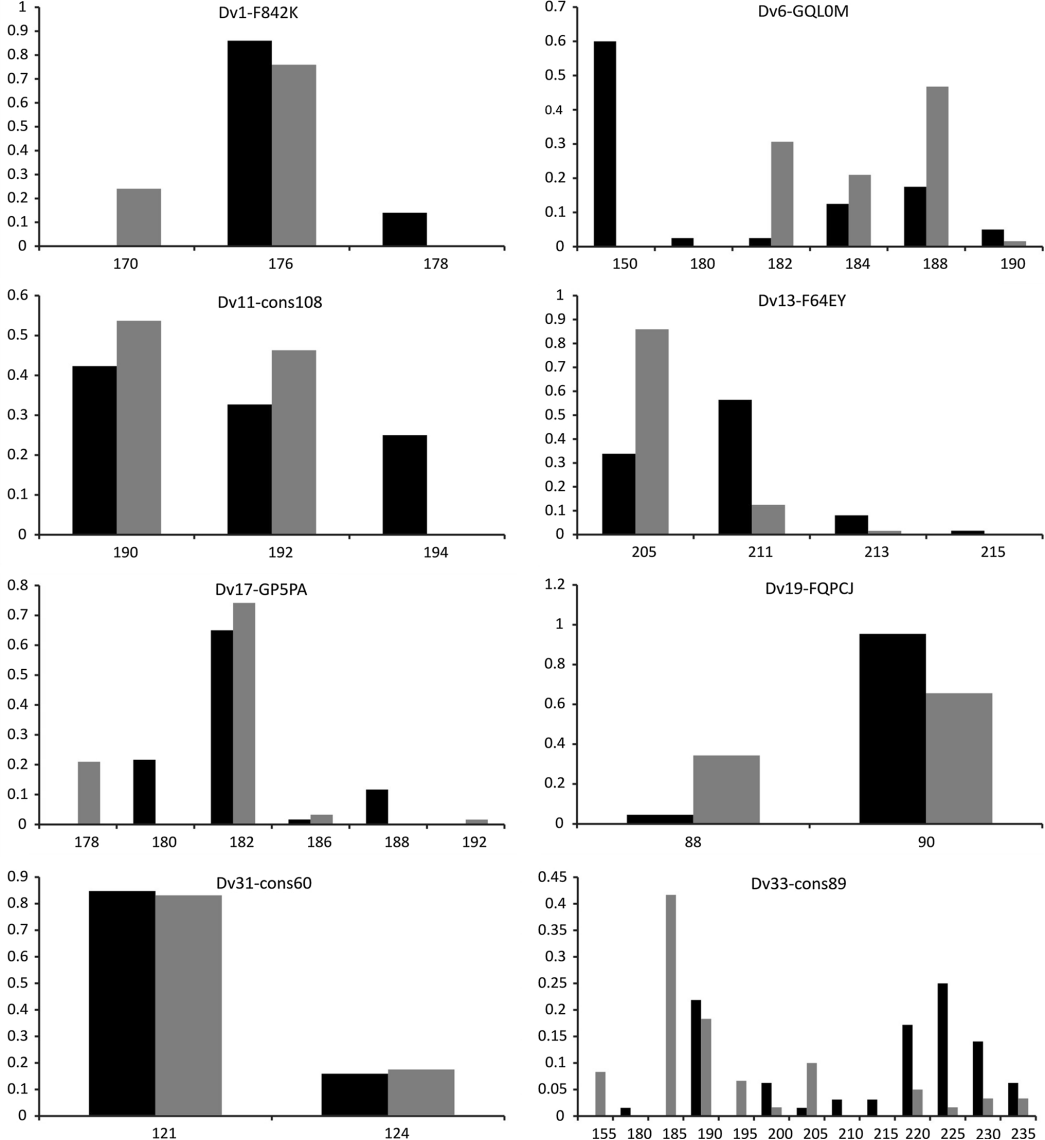
Allele frequency distribution for each locus for the DAN (*black*) and DNI (*grey*) populations. *Axis x* allele size in bp, *axis y* frequency of alleles

Based on the 64 genotyped individuals from the Danube (DAN) and the Dnieper (DNI) populations, we obtained a mean diversity of 4.87 alleles per locus, ranging from 2 to 13 (Table 1). The mean observed and expected heterozygosities were, respectively, 0.439 (0.091–0.844) and 0.468 (0.089–0.843). The Fstat software detected neither the gametic disequilibrium for any pair of loci, nor a deviation from the Hardy–Weinberg proportions in any locus in any of the two populations. However, Micro-checker detected sign of a null allele at Dv1 in both DAN and DNI and at Dv6 for DNI only. DAN and DNI populations were differentiated with a significant Fst value of 0.17. Although the invasion dynamics of the killer shrimp along the Danube and in French rivers was assessed by Wattier et al. [19] based on the three microsatellite loci available at that time [18], additional loci are needed for further assessment of its expansion all over Europe. The eight new loci will be highly valuable in identifying sources of introduction for the Alpine lakes and for the UK, that are not directly connected to any of the invasion highways (Fig. 1). The differentiation between DAN and DNI populations illustrates that such source populations could be relatively easily identified with a higher number of loci. Moreover, these markers could help to detect possible hybridization and/or introgression between the two populations of *D. villosus* which may become in contact in Poland [26].

Finally, it is known that microsatellite markers characterized for one species may often reveal polymorphism in other closely related taxa [27]. Thus we suggest that the loci described here have potential to be amplified in species closely related to the “killer shrimp” such as *Dikerogammarus haemobaphes* (Eichwald, 1841) and *Dikerogammarus bispinosus* Martynov, 1925 which are also invasive in European inland waters [28] and, in case of the latter, also in the UK. [29].
